# Deep Sea Water Prevents Balloon Angioplasty-Induced Hyperplasia through MMP-2: An *In Vitro* and *In Vivo* Study

**DOI:** 10.1371/journal.pone.0096927

**Published:** 2014-05-13

**Authors:** Pei-Chuan Li, Chun-Hsu Pan, Ming-Jyh Sheu, Chin-Ching Wu, Wei-Fen Ma, Chieh-Hsi Wu

**Affiliations:** 1 School of Pharmacy, China Medical University, Taichung, Taiwan; 2 College of Pharmacy, Taipei Medical University, Taipei, Taiwan; 3 Department of Public Health, China Medical University, Taichung, Taiwan; 4 School of Nursing, China Medical University, Taichung, Taiwan; 5 Department of Biological Science and Technology, China Medical University, Taichung, Taiwan; University of Iowa, United States of America

## Abstract

Major facts about the development of restenosis include vascular smooth muscle cells (VSMCs) proliferation and migration. A previous study showed that in vitro treatment with magnesium chloride has the potential to affect the proliferation and migration of VSMCs. Magnesium is the major element in deep sea water (DSW) and is a biologically active mineral. It is unclear whether DSW intake can prevent abnormal proliferation and migration of VSMCs as well as balloon angioplasty-induced neointimal hyperplasia. Thus, we attempted to evaluate the anti-restenotic effects of DSW and its possible molecular mechanisms. Several concentrations of DSW, based on the dietary recommendations (RDA) for magnesium, were applied to a model of balloon angioplasty in SD rats. The results showed that DSW intake markedly increased magnesium content within the vascular wall and reduced the development of neointimal hyperplasia. The immunohistochemical analysis also showed that the expression of proteins associated with cell proliferation and migration were decreased in the balloon angioplasty groups with DSW supplement. Furthermore, in vitro treatment with DSW has a dose-dependent inhibitory effect on serum-stimulated proliferation and migration of VSMCs, whose effects might be mediated by modulation of mitogen-activated protein kinase (MAPK) signaling and of the activity of matrix metalloproteinase-2 (MMP-2). Our study suggested that DSW intake can help prevent neointimal hyperplasia (or restenosis), whose effects may be partially regulated by magnesium and other minerals.

## Introduction

As a result of balloon injury-induced restenosis, VSMCs predominantly undergoes proliferation and migration [1].The restenotic process is initiated by balloon angioplasty-induced vascular injury, which stimulates VSMCs to migrate from the medial layer to the intimal layer of the vessel wall and eventually results in uncontrolled neointimal hyperplasia. Accordingly, to inhibit proliferation and migration of VSMCs is valuable of reducing balloon injury-induced angioplasty.

The major problem of mineral deficiencies caused by the recent westernization of lifestyles and eating habits has resulted in so-called lifestyle-related illnesses such as coronary heart disease, angina pectoris, myocardial infarct, stroke, cancer, and diabetes [2], [3]. Moreover, Mg^2+^ is hypothesized to play an important role as a protective element of cardiovascular diseases. [4], And, intake of Mg^2+^ has been linked with reduction of blood pressure, triglyceride, and arrhythmia from congestive heart failure patients [5]. Recently, Sternberg *et al*. indicated that appropriate concentration of magnesium chloride has a potential in****vitro effect to reduce cell viability of the primary VSMCs while increasing the viability of endothelial cells isolated from human coronary artery [6]. Additionally, the experimental analysis from a cDNA microarray also demonstrated that numerous genes for growth factors and their receptors, as well as for cell cycle and apoptosis-related signaling cascades, have been markedly up- or down regulated within these magnesium-chloride-treated VSMCs compared to those of endothelial cells [6]. Mg^2+^ deficiency has been associated with hypertrophic vascular remodeling, however, this phenomenon could be attenuated by Mg^2+^ supplementation [7]. From abovementioned results, Mg^2+^ deficiency might attribute to the development of hypertension, atherosclerosis, and other cardiovascular diseases (CVD).

Recently, deep sea water (DSW) has received increasing attention for the treatment or prevention of various diseases, such as hyperlipidemia, atherosclerosis, hypertension, and dermatitis [8]–[11]. DSW is characteristically clear, sanitary, and plentiful in nutrients, being especially rich in Mg^2+^, Ca^2+^ and K^+^. [8], [12]. In this study, we investigate if DSW is of beneficial to prevent balloon angioplasty-induced neointima hyperplasia and its possible pharmacological mechanisms.

## Materials and Methods

### Materials and DSW Preparation

The DSW was obtained from the Pacific Ocean (662****m in depth) and was then concentrated. This concentrated DSW (#LC-90K) with a hardness of 400,000****mg/L was supplied by Taiwan Yes Deep Ocean Water Co., Ltd. (Hualien, Taiwan) The DSW composition contained Mg^2+^, K^+^, Ca^2+^, sodium (Na^2+^), chloride (Cl**^−^**), lithium (Li^+^), and other trace elements. In this experiment, the Mg^2+^ content in DSW was 96,000****mg/L, as mentioned in our previous study [8].

### Balloon Injury Animal Model

Male Sprague Dawley (SD) rats (250**∼**300****g) were bought from BioLASCO Taiwan Co. Ltd (Taipei, Taiwan). Surgery was performed under zoletil anesthesia. The rats were randomly divided into six groups (n = 6/group): (A) sham control+water, (B) balloon angioplasty (BA)+water, (C) BA+37.2****mg Mg^2+^/kg/day (MgCl_2_), (D) BA+0.1x DSW (equivalent to 3.72****mg Mg^2+^/kg/day), (E) BA+1x DSW (equivalent to 37.2****mg Mg^2+^/kg/day), and (F) BA+2x DSW (equivalent to 74.4****mg Mg^2+^/kg/day).Reverse osmotic (RO) water was considered as water administration in groups A and B. The magnesium RDA for adult males is 360****mg/day (6****mg/kg/day); thus, the dosage of Mg^2+^ for SD rats is 37.2****mg Mg^2+^/kg/day conversed by using a U.S. FDA dose conversion table, human equivalent dose. The dosages of DSW applied in the present study were calculated as the equivalent content of Mg^2+^ RDA for rats. Therefore, 1x DSW was defined as water-diluted DSW containing 37.2****mg/kg of Mg^2+^. The rats were given DSW for 4 weeks and performance angioplasty during the second week. Balloon injury-induced neointimal hyperplasia was carried out using a balloon embolectomy catheter as described previously [13] A Fogarty 2F (Becton-Dickinson, Franklin Lakes, NJ, USA) embolectomy balloon catheter was inserted into the left external carotid artery and inflated under the same pressure (1.3****kg/cm^2^) for three consecutive three times. All animal care followed the institutional animal ethical guidelines of China Medical University. The experimental protocol was approved by the Committee on Animals Research, China Medical University (Permit Number: 100-49N).

### Histopathological Analysis

The left carotid artery was excised and fixed in 4% paraformaldehyde solution for 24****h, processed for paraffin embedding, and cut into 7-mm transverse sections. The tissue sections were stained with a routine H&E staining. Five random sections of each studied sample were randomly selected and measured with image analysis software (Image J) to calculate the neointima-to-media area ratio (N/M ratio).

For immunohistochemical analysis, the DAKO system (#K0679; Dako LSAB+System-HRP, DAKO, Tokyo, Japan) was used. It employs a refined avidin-biotin technique in which a biotinylated secondary antibody reacts with several peroxidase-conjugated streptavidin molecules. The tissue sections were rehydrated and immersed in 3% H_2_O_2_ for 30****minutes to quench endogenous peroxidase, and then all sections were further incubated in 1% bovine serum albumin (BSA; Sigma Aldrich, USA) for 1****h at 25**°**C. Next, the sections were incubated with a primary antibody against either matrix metalloproteinase-2 (MMP-2; #ab37150; Abcam, USA) or proliferating cell nuclear antigen (PCNA; #sc-56; Santa Cruz, USA) overnight at 4°C. Then, the sections were incubated for 30****min at room temperature with the biotinylated link antibody and peroxidase-labeled streptavidin. For signal detection, the sections were incubated with the ready-to-use DAB substrate-chromogen solution for 5****min according to the manufacturer’s protocol and then washed with distilled water. Finally, sections were counterstained with hematoxylin for 3****min, followed by washing with distilled water and mounting with a hard-set media (Assistant-Histokitt, Germany). Negative controls consisted of primary antibody replaced with buffer-specific antibody absorbed with antigen. Photomicrographs were taken with a microscope (Olympus, Japan) at 200-fold magnification.

### Blood Biochemical Assay

The serum was collected in non-heparinized tubes and centrifuged at 3000 rpm for 10 min at 4°C. The blood biochemical parameters including alanine aminotransferase (ALT), aspartate aminotransferase (AST), alkaline phosphatase (ALP), γ-glutamyltransferase (GGT), and the serum concentration of Mg^2+^ were measured by ChenChang Co., Ltd. (Taichung, Taiwan).

### Measurement of Arterial Magnesium Content

The harvested arterial tissues were rinsed with deionized water and dried in 60°C for 24****h, followed by measuring tissue weight. Approximately 0.5****g of dried tissue was further digested with 5****mL of 65% HNO_3_ (E. Merck, Darmstadt, Germany). The determine sample was diluted to 10****mL with deionized water. We measured the tissue Mg^2+^ levels with quadruple inductively coupled plasma mass spectrometry (ICP-MS) (Elan DRC II, Perkin Elmer, USA), which is capable of detecting metals and several non-metals at concentrations as low as one part per trillion (ppt). The sample is typically introduced into the ICP plasma as an aerosol, either by aspirating a liquid or by using a laser to directly convert solid samples into an aerosol, and the elements are converted into ions that are then brought into the mass spectrometer via the interface cones [14].

### Cell Culture

VSMCs were prepared from thoracic aortas of 4–6-week old male SD rats. Cells at passages 3–6 were applied for the present study. The cells were cultured in Dulbecco’s modified Eagle’s medium (DMEM) with 10% FBS, 100 units/L penicillin, and 100****mg/L streptomycin. The cells were maintained at 37°C in a humidified 5% CO_2_ incubator.

### Cell Proliferation Assay (MTT Assay)

VSMCs were seeded and cultured in 96-well plates (8×10^3^ cells/well) for 24****h. VSMCs were then treated with MgCl_2_ or different concentration of DSW for 24****h. For in****vitro experiments, MgCl_2_ or different concentration of DSW were added to medium containing 15% FBS. MTT assay was used to determine VSMCs proliferation: 100** µ**l of 3-(4, 5-Dimethylthiazol-2-yl)-2, 5-Diphenyltetrazolium Bromide (MTT) (0.5****mg/ml) was added to each well and incubated at 37°C for 4****h, after which the supernatant was removed and replaced with DMSO (100** µ**l). The product was quantified by measuring absorbance at 590****nm. The cell viability was normalized to the values from the group that received 15% FBS alone.

### Cell Migration Assay (Transwell Assay)

Cell migration of VSMCs (5×10^3^ cells/well) was analyzed with wound-healing and transwell assays in 24-well multiwall plates (8.0** µ**m pore-size, Greiner bio-one, USA) for 24****h. The cell were loaded into the upper compartment and incubated for 18****h. Afterward, the cells were collected from the lower membrane and fixed with 70% methanol. VSMCs were stained with Giemsa and hematoxylin solutions and then counted under a microscope (Olympus, Japan) in five randomly selected squares per well. The cell migration was normalized to the values of the 15% FBS group.

### Western Blotting Assay

Cells were lysed in PRO-PREP protein extraction solution (iNtRON Biotechnology, Sungnam, South Korea). Protein samples (30** µ**g/well) were electrophoresed via 10% SDS-PAGE. Gels were transferred onto polyvinylidene difluoride (PVDF) membranes (BioTrace™, USA). Then, blotted membranes were blocked in 5% non-fat milk for 1****h and incubated overnight at 4°C with the primary antibodies against phospho-mitogen-activated protein kinase (p-MEK1/2) (#9121; Cell Signaling, UK.), extracellular signal-regulated kinase 1/2 (p-ERK 1/2) (#sc7383; Santa Cruz Biotechnology, U.S.A.), or matrix metalloproteinase-2 (MMP-2) (#ab37150; Abcam, U.S.A.). After incubation with appropriate secondary antibodies (Gene Tex, U.S.A.) for 1****h, blot were incubated with enhanced chemiluminescent (ECL) reagent, and the images were acquired using an ImageQuant LAS4000 gel imager (Fujifilm Life Science, Tokyo, Japan). Data were quantified by densitometry using Multi Gauge v 3.0 software (Fujifilm Life Science, Tokyo, Japan). The protein expression was normalized to β-actin and 15% FBS group was considered as 100%.

### Gelatinase Zymography

The culture medium was harvested to examine gelatinase activity via 10% SDS–PAGE gel electrophoresis with 0.2% gelatin under non-reducing conditions. After electrophoresis, gelatinases were renatured by rinsing the gel in 2.5% Triton X-100 at 25°C for 30****min, and then activated in reaction buffer (2 M Tris-HCl, pH 8.0, 1 M CaCl_2_, and 1% NaN_3_) at 37°C for 24****h. The gels were stained with 0.25% Coomassie brilliant blue R250 for 90****min and destained with 10% acetic acid in 40% methanol. Gelatinase activity was evident as a clear band against the blue background of stained gelatin. Bands were quantified by densitometry using Multi Gauge v3.0 software. The MMP-2 activity was normalized to 15% FBS group.

### Data Analysis

Experiments data were presented as the mean ± SEM. Data were analyzed using one-way ANOVA. Calculations were carried out using SPSS for Windows, version 18.0 (SPSS, Chicago, USA).Differences were considered significant at *P*<0.05.

## Results

### Changes of Blood Biochemical Parameters and Body Weight

SD rats were grouped into six experimental conditions, as described in the Experimental Methods. All rats were weighed and blood samples were taken once a week during the 4-week study. Numerous blood biochemical parameters including ALT, AST, ALP, and GGT, and the serum concentration of Mg^2+^ were examined and shown in Table 1. Our data showed that ALP level was significantly increased on BA + water group, however, DSW-treated groups reversed the results (Table 1). Our results shown no dramatic change of the serum Mg^2+^ concentration between DSW-treated and sham + water group (Table 1). Furthermore, no significant differences were found between the DSW-treated (0.1x, 1x and 2x DSW) and control groups (sham + water or BA + water group) in body weights after 4-week study (Data not shown). Our results suggested the dosages of DSW had no marked toxic effects, such as hepatotoxicity.

**Table 1:** Effect of blood biochemical parameters of SD rats after 4-week treatment with sham + water, BA + water, BA + MgCl_2_, and DSW.

GroupsParameters	Sham	BA	BA	BA	BA	BA
	water	water	MgCl_2_	0.1x DSW	1x DSW	2x DSW
**ALT**	58.0±8.9	49.5±2.9	49.50±3.6	42.7±3.4	50.8±6.2	49.0±8.9
**AST**	152.7±51.2	123.3±19.1	103.8±12.5	114.8±23.6	115.0±9.2	104.8±20.4
**ALP**	573.3±36.8	877.2±17.9*	765.0±19.9*	506.3±45.6	657.8±11.9	778.5±19.4*
**GGT**	0.6±0.2	0.9±0.9	1.6±0.3	0.5±0.3	0.57±0.3	1.1±0.6
**Initial Mg^2+^**	21.9±2.4	24.5±4.2	21.1±0.9	22.4±2.1	23.4±2.6	24.3±1.8
**Final Mg^2+^**	23.1±2.0	22.5±2.7	23.0±1.2	20.7±1.8	20.7±1.4	20.0±2.5

Concentration unit: U/L. BA, balloon angioplasty; RDA, recommended dietary allowances; ALT, alanine aminotransferase; AST, aspartate aminotransferase; ALP, alkaline phosphatase; GGT, γ-glutamyltransferase. Mg^2+^: serum Mg^2+^ concentration. Values are mean ± SEM, n = 6.

*p<0.05 compared to sham + water.

### Effect of DSW on Balloon Angioplasty-induced Neointimal Hyperplasia

**Figure 1:**
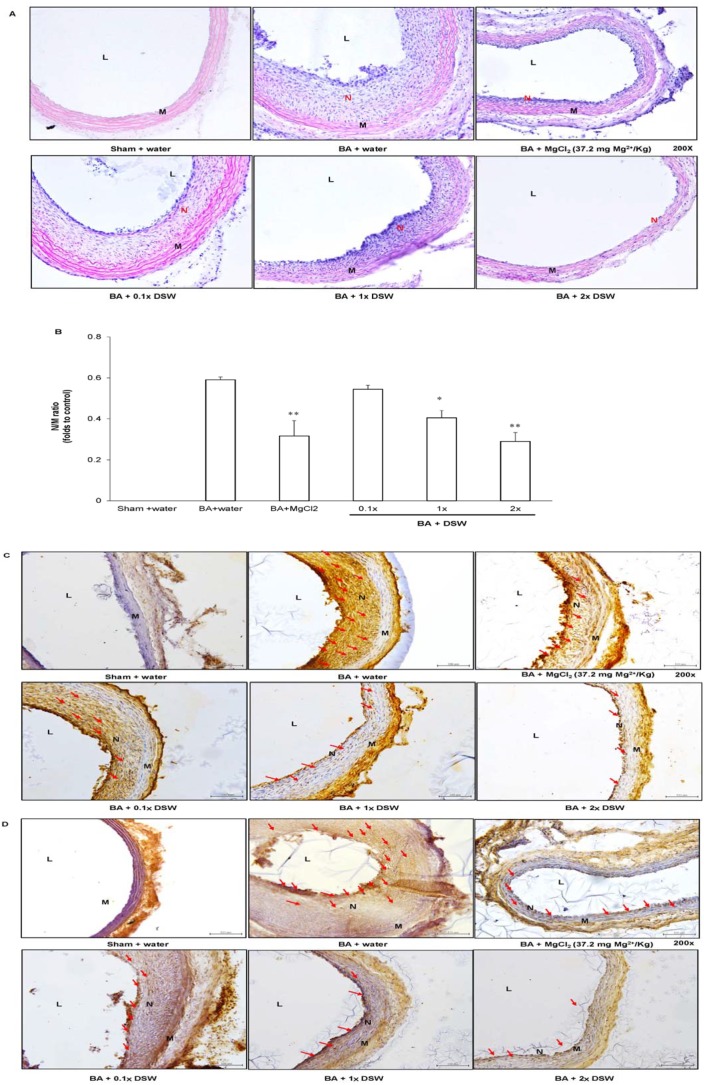
Inhibitory effect of DSW on balloon angioplasty-induced neointimal hyperplasia. Tissue sections from rat carotid arteries were further stained with hematoxylin-eosin to observe the thickness of neointimal layer of arterial wall (A–F). The expression levels of MMP-2 and PCNA proteins were detected with immunohistopathological analysis (H and I). The images were acquired by microscopy at 200-fold magnification. The manifestation of vessel restenosis was presented as the ratio of neointima- to-media area (N/M ratio). L, lumen; N, neointima layer; M, media layer. Red arrow is protein experssion.*P<0.05, and **P< compared with BA + water group, respectively.

The rat carotid arteries were harvested for examination of the histopathological changes in the arterial wall at the 14^th^ day after balloon injury (Figure 1). The balloon angioplasty procedure (BA + water group; Figure 1B) induced significant induced neointimal hyperplasia compared to the sham control (Sham + water group; Figure 1A). The N/M ratio of the positive control (BA + water group) was increased more than 10-fold over that of the sham group (Figure 1G). However, the groups co-treated with DSW (Figure 1D–1F) possessed markedly less development of neointima formation, in a dose-dependent pattern (Figure 1G). Similarly, MgCl_2_ treatment also reduced balloon injury-induced neointimal hyperplasia (Figures 1C and 1G). These results demonstrated that DSW intake exhibits an inhibitory effect on the prevention of neointimal hyperplasia, implying that ionic magnesium may participate in some of the mechanisms of DSW on prevention of neointima formation. The sections were also evaluated by immunohistochemistry to detect MMP 2 (MMP-2; Figure 1H), a cell migration and invasion-associated protein, and PCNA (PCNA; Figure 1I), a cell proliferation marker. MMP-2 expression was visibly induced in the neointimal and media layers after balloon angioplasty, and pretreatment with DSW (1x and 2x DSW) and MgCl_2_ could reduce the expression of MMP-2 within the vessel wall. A similar trend was visible for PCNA expression after supplementation with DSW or MgCl_2_.

### Magnesium Content of the Carotid Artery

**Figure 2:**
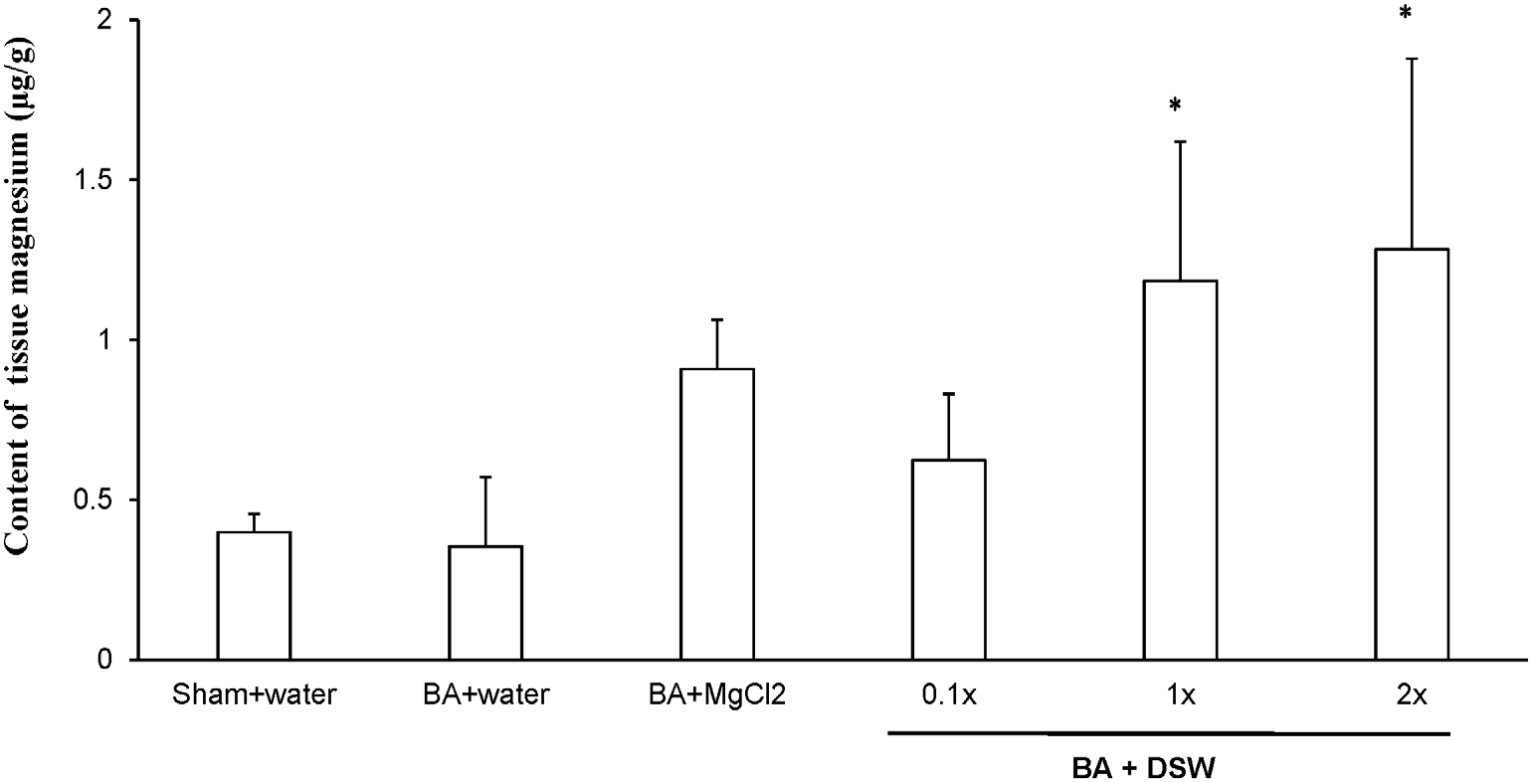
Differences in magnesium content in the balloon-injured carotid artery. *P<0.05 compared with BA + water group, respectively.

The rat carotid arteries were harvested to examine the changes of Mg^2+^ content within arterial wall at the 14^th^ day after balloon injury (Figure 2). Mg^2+^ content of the tissue samples were measured by quadruple ICP-MS. DSW intake clearly increased the magnesium content within balloon-injured arteries in a dose-dependent manner when compared to the BA + water group. However, the level of serum Mg^2+^ did not significantly change in any group in the present study (data not shown). Ma *et al.* showed a negative association between serum Mg^2+^ and average carotid wall thickness [15]. Pascal *et al.* have shown that Mg^2+^ deficiency modifies the mechanical properties of the common carotid artery, thereafter attribute to cardiovascular diseases (CVD) development in animal models [16]. They also indicated that long-term oral supplementation of magnesium improves blood lipid composition and suppresses the development of atherosclerotic lesions in rodents [17], [18]. In contrast, Ferre *et al.* showed that low Mg^2+^ accelerated atherogenesis by stimulating inflammation and oxidative stress in endothelial cells, suggesting a possible mechanism linked to CVD [19]. Wolf *et al.* also reported that Mg^2+^ deficiency could be associated with inflammatory responses resulting into the increased cytokines levels, which cause an oxidative damage in endothelial cells [20]. Especially, low intracellular Mg^2+^ may be linked with the development of thrombus formation [21].

### Effects of DSW on VSMC Proliferation and Migration

**Figure 3:**
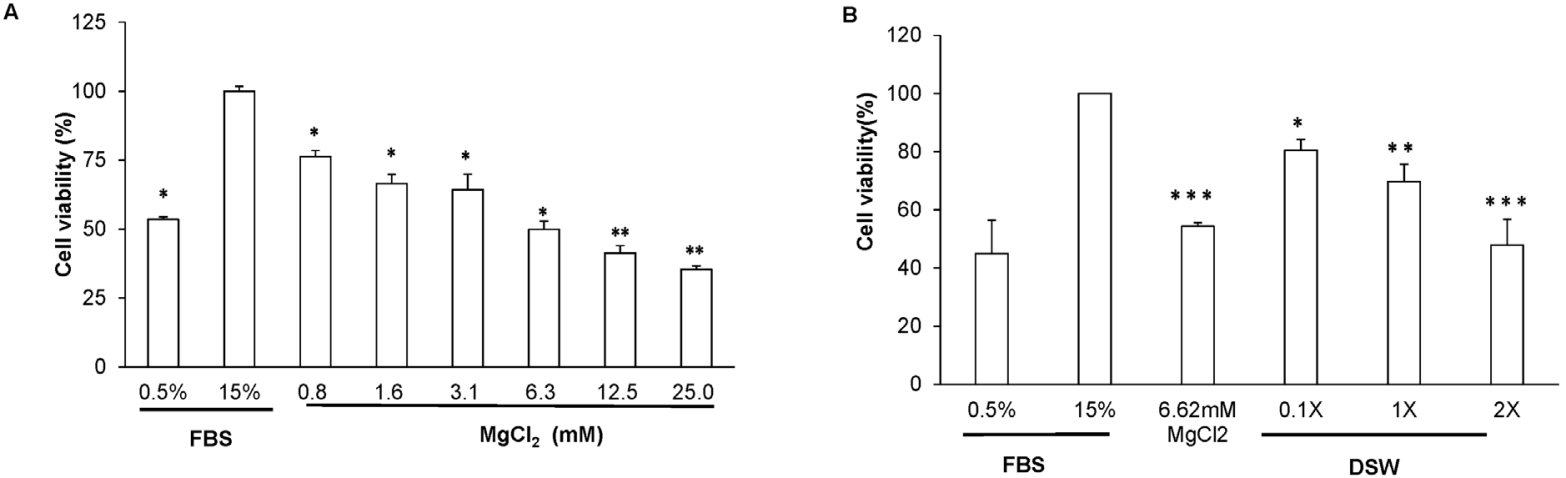
DSW-induced regulation of VSMC viability. Cell viability was analyzed with the MTT proliferation assay. One-fold (1x) DSW was defined as water-diluted DSW containing a level of magnesium equal to that of the magnesium chloride used in the present experiment. The results are shown as % of the control. *P<0.05, and **P<0.01 compared with the 15% FBS-treated group, respectively.

VSMCs isolated from rat thoracic aorta were used to determine the effects of DSW on cell proliferation and migration. First, magnesium chloride was applied, to verify that it inhibits VSMC growth. We found that 15% FBS can significantly stimulate cell proliferation of VSMCs when compared with the low serum stimulation (0.5% FBS) group, and treatment of magnesium chloride can block half of the VSMC proliferation at the concentration of 6.62 mM (Figure 3A). Next, the effects of DSW on cell growth of VSMCs were examined. DSW treatment could markedly decrease cell viability in a dose-dependent manner (Figure 3B). The 1x DSW and MgCl_2_ groups showed similar growth inhibition on VSMCs. However, treatment with 2x DSW was more inhibitory toward cell growth than was the MgCl_2_ treatment (*P*<0.05).

**Figure 4:**
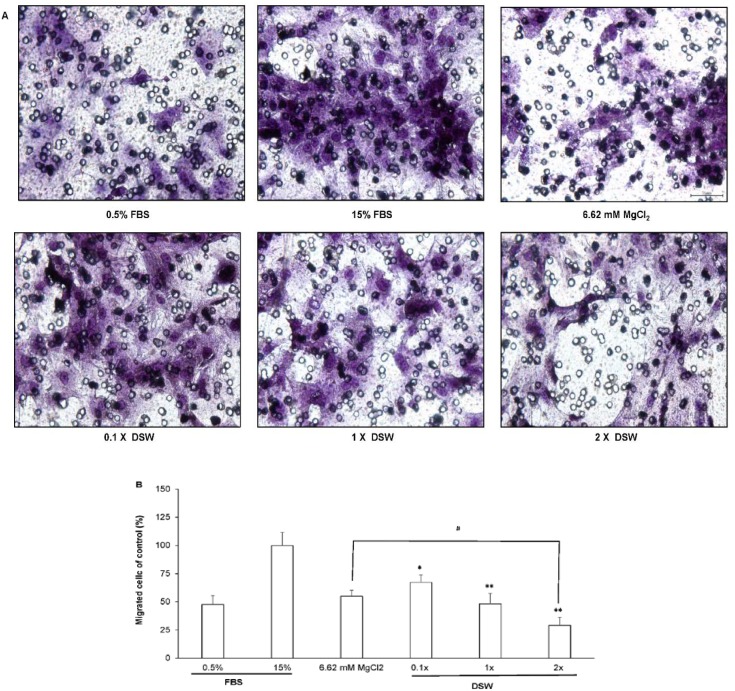
Effects of DSW on VSMC migration. Cell migration was analyzed with transwell assays. The images were taken at 400-fold magnification. The 1x DSW was defined as water-diluted DSW containing the same level of magnesium as the magnesium chloride used in the present experiment. The results are shown as % of the control (15% FBS).*P<0.05, and **P<0.01 compared with the 15% FBS-treated group, respectively. #*P*<0.05 compared with the MgCl_2_-treated group.

The transwell assay was used to explore the effect of DSW treatment on VSMC migration (Figure 4). High serum stimulation (15% FBS) can induce cell migration of VSMCs (Figure 1B) compared with the 0.5% FBS group (Figure 1A), and this migration-promoting effect can be markedly attenuated by incubation with DSW, in a dose-dependent manner (Figure 1D–1F). Administration with magnesium chloride showed a similar reduction of serum-stimulated VSMC migration (Figure 1C).

### DSW Inhibits Proliferation and Migration-associated Proteins on VSMCs

**Figure 5:**
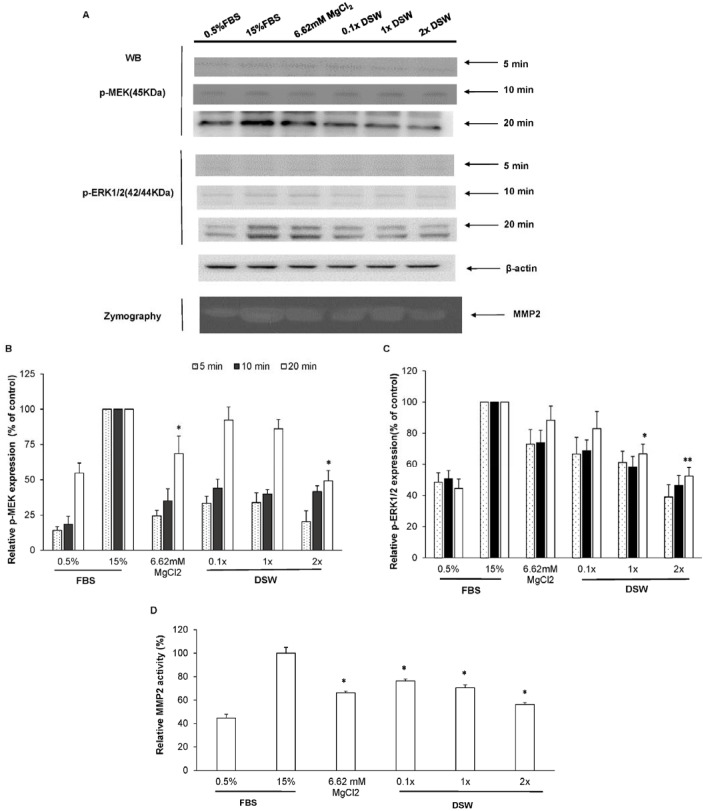
Molecular regulation by DSW of cell growth and migration of VSMCs. Inhibitory mechanisms of DSW on cell growth- and migration-associated proteins were analyzed by using the methods of Western blot (WB) and gelatin zymography. Beta-actin was used as internal control in Western blot. The results are shown as % of the control (15% FBS). The MMP-2 activity was normalized to the values of the 15% FBS group. *P<0.05, and **P<0.01 compared with the 15% FBS-treated group, respectively.

MAPK signaling pathways are associated with cardiovascular disease [22].The signaling of the MAPK pathway plays an important role in the regulation of VSMC proliferation [23]. The first member of this family was ERK1/2, which is activated after MEK1/2 phosphorylation. It has been reported that ERK1/2 activation is rapidly induced after arterial injury and might trigger a series of molecular events leading to neointima hyperplasia [24]. The inhibition of the ERK pathway by drugs or gene therapy has been demonstrated to reduce neointimal hyperplasia [25]. Phosphorylation MEK and ERK1/2 were both examined at multiple time points including 5 min., 10 min., and 20 min. And the levels of phosphorylation MEK and ERK1/2 showed obvious changes at 20 min (Figure 5A–5C). DSW inhibited ERK phosphorylation and MEK phosphorylation, which are associated with VSMC proliferation and migration (Figures 5A, 5B and 5C). Besides, MMPs is evident to regulate the migration activity of VSMCs in *in vitro* and *in vivo* studies [26]. The results from gelatin zymography suggested that high-dose DSW decreased MMP-2 activity by half compared with that from the 15% FBS group (Figures 5A and 5D), implying that the DSW-induced inhibitory effect on the migration of VSMCs may be partially mediated by decreased MMP-2 activity.

Our results shown that DSW intake inhibits VSMCs proliferation and migration in addition to its anti-restenosis effect in *in vivo* study.

## Discussion and Conclusions

As a rich source of inorganic nutrients such as Mg^2+^, Ca^2+^, and other minerals (K^+^, Cu^2+^, Mn^2+^, Zn^2+^, B^−^, and Li^+^), DSW in particular hold the attention in CVD studies.

As a Ca^2+^ channel blocker, Mg^2+^ is a key role regulating the reduction of Ca^2+^ release and thus reduce vascular resistance [7]. Fruits and vegetables, diets rich in K^+^, Mg^2+^, and Ca^2+^ is associated with lower incidence of and mortality of CVD [27]. Moreover, Turgut *et al.* reported that supplementation of Mg^2+^ can efficiently reduce the carotid intimal hyperplasia [28]. Mg^2+^ has been found to have many effects, including improving hyperlipidemia and preventing the development of atherosclerosis [12], [29]. Patients with hypertension or hyperlipidemia orally supplied with calcium or Mg^2+^ showed reductions in blood pressure and serum levels of total cholesterol [30].

DSW has shown an inhibitory effect on serum-stimulated proliferation and migration of VSMCs *via* inhibition of ERK1/2-MAPK signaling and MMP-2 activation (Figure 5). Based on our previous report [8]. Mg^2+^ is found to be the most abundant ingredient in DSW. Mg^2+^ regulates a MAPK signaling cascade, which is associated with VSMCs cell growth, cell division, migration, and proliferation [6], [31]. Accordingly, our data showed no change on intracellular Mg^2+^ (Table 1); however, tissues Mg^2+^ concentration has been significantly increased after DSW supplementation (Figure 2). Touyz *et.al*. showing modulation of cell growth by Mg^2+^ was due to influx/efflux Mg^2+^ and regulation of MEK and ERK1/2 [32]. Intracellular Mg^2+^ is delicately regulated in the physiological condition and because Mg^2+^ plays important roles in multiple physiological reactions, even small changes in VSMCs, markedly affects intracellular signaling transduction regulating VSMCs [3], [32].

Our data demonstrated that MgCl_2_ only slightly regulated phosphorylation MEK, with no effects on phosphorylation ERK1/2 (Figure 5). However, DSW significantly affect the MAPK signaling molecules including MEK and ERK1/2 (Figure 5). Therefore, minerals other than MgCl_2_ in DSW such as Cu^2+^, Zn^2+^, Li^+^, B^−^ and Mn in DSW might have certain effects on VSMCs proliferation and migration *via* regulating MEK and ERK1/2. Cu/Zn-superoxide dismutase (SOD) and Mn-SOD were found to play important roles in scavenging of superoxide [33]. Zhuyao *et al.* also reported that lithium chloride inhibited VSMCs proliferation, migration and alleviated balloon injury-induced angioplasty [34]. Furthermore, Nielsen *et al.* reported that boron prevents Mg^2+^ loss in humans [19]. Therefore, Mg^2+^ may not be sole strategy of dealing injury from balloon angioplasty. However, DSW is a well-balanced mineral resource, and dietary supplementation with DSW provides more beneficial functions than magnesium chloride alone in the prevention of cardiovascular diseases, such as restenosis and atherogenesis.

As DSW will be applied for the nutrition supplement, therefore, the toxic effect has to be concerned. Our results showed that levels of ALT, and AST on DSW-treated groups had no significant change compared to Sham + water or BA + water groups (Table 1). Previously, Sheu *et.al.* reported that drinking DSW for a long period (56 days; 8 weeks) demonstrated no liver toxicity in rats and rabbits [8]. Fu *et.al.* showed that fed a 6 months DSW diet decreased serum total and low-density lipoprotein in a human study [35]. Based on the abovementioned, DSW could be a potential functional supplement to prevent balloon angioplasty-induced neointimal formation.
